# Patients’ Perspectives on Bedside Manners in Physical Therapy in Saudi Arabia: A Mixed-Methods Study

**DOI:** 10.3390/healthcare14193231

**Published:** 2026-10-01

**Authors:** Saleh M. Aloraini, Faris M. Aba-Alkhayl, Sara H. Althinayyan, Moodhi M. Alfouzan, Fayzah A. Almohaimeed, Alanoud I. Alsughayyir, Raghad Aljutaily, Ruba S. Alquraishi, Renad K. Aldughaim, Ryhana M. Alnoshan, Jana B. Albarrak, Wasan A. Alsaheel, Fai A. Alqazlan, Ali Alsouhibani

**Affiliations:** 1 Department of Physical Therapy, College of Applied Medical Sciences, Qassim University, Buraydah 51452, Saudi Arabia; pt.sarathinayyan@gmail.com (S.H.A.); modyalfowzan@gmail.com (M.M.A.); ra.aljutaily@gmail.com (R.A.); rubaalquraishi22@gmail.com (R.S.A.); renadaldughaim@gmail.com (R.K.A.); a.alsouhibani@qu.edu.sa (A.A.); 2 Department of Occupational Therapy, College of Medical Rehabilitation Sciences, Taibah University, Madinah 42353, Saudi Arabia; fabaalkhayl@taibahu.edu.sa; 3 Kheraif Physical Therapy Clinic, Buraydah 52387, Saudi Arabia; fayzahazizz@gmail.com (F.A.A.); janaalbarrak@gmail.com (J.B.A.); 4 Rehabilitation Programs and Services Department, Sultan Bin Abdulaziz Humanitarian City, Riyadh 11536, Saudi Arabia; nmalanoud@gmail.com (A.I.A.); ryhana.alnoshan@gmail.com (R.M.A.); 5 Qassim Health Cluster, Buraydah 52366, Saudi Arabia; walsaheel@moh.gov.sa; 6 Rehabilitation Department, Ministry of the National Guard-Health Affairs, Riyadh 11426, Saudi Arabia; fay3li.fa@gmail.com

**Keywords:** bedside manners, physical therapy, patient care, mixed-methods, ethics

## Abstract

**Highlights:**

**What are the main findings?**

- Patients reported highly positive experiences with core bedside-manner behaviors, while preferences regarding empathy and therapist supervision were more variable.
- Preferences for therapist presence differed by therapist sex and patient–therapist sex match, although effect sizes were small.

**What are the implications of the main findings?**

- Physical therapists should maintain strong communication, privacy, and consent practices while giving greater attention to shared decision-making and individual patient preferences.
- Physical therapy education should promote individualized, patient-centered, and culturally responsive communication and supervision.

**Abstract:**

**Background/Objectives**: Bedside manners are central to patient-centered rehabilitation, yet patient perspectives on physical therapy in Saudi Arabia remain underexplored. This study examined patients’ perceptions of physical therapists’ bedside manners and explored variation according to patient and therapist characteristics. **Methods**: A cross-sectional convergent mixed-methods study included 510 patients who had completed physical therapy within the previous year. Participants completed a 25-item questionnaire comprising six sociodemographic and 19 bedside-manner items; 25 participants also completed semi-structured interviews. Quantitative data were summarized descriptively, and selected Likert-scale items with sufficient response variability were examined using chi-square or Fisher’s exact tests with Bonferroni correction and Cramér’s V. Interview data underwent thematic analysis. **Results**: Responses to the nine yes/no items were predominantly affirmative (88.6–98.6%). The first item assessed familiarity with the term “bedside manners,” whereas the remaining eight items assessed patients’ reported experiences of therapist behaviors. Agreement exceeded 90% for six of the ten Likert-scale items, while responses regarding empathy, therapist presence, and attention according to case complexity were more variable. After correction for multiple comparisons, preferences regarding therapist presence differed by therapist sex and patient–therapist sex match (both *p* < 0.001; Cramér’s V = 0.17). The qualitative analysis identified seven themes: therapeutic relationship, treatment adherence, nonverbal communication, emotional support, therapist supervision, initial impression, and professional consultation. **Conclusions**: Patients in this sample strongly valued communication, consent, privacy, reassurance, and respectful interpersonal behavior, while preferences regarding empathy and therapist supervision were more individualized. Interpretation is limited by the cross-sectional convenience sample, restriction to treatment completers, and the small magnitude of the observed associations.

## 1. Introduction

Effective healthcare extends beyond technical competence to incorporate humanistic, ethical, and interpersonal dimensions [1]. The concept of bedside manners is increasingly recognized as a critical component of patient-centered care [1]. Abuqayyas et al. [1] defined bedside manners as encompassing all aspects of patient interaction, including both verbal and nonverbal communication strategies. In the present study, bedside manners are conceptualized as an umbrella term encompassing verbal, nonverbal, ethical, and interpersonal behaviors during clinical interactions, including communication, respect, privacy, consent, reassurance, empathy, and professional conduct [1]. Related concepts such as empathy and the therapeutic relationship are therefore considered components of, or closely related to, bedside manners rather than synonymous terms. This comprehensive approach highlights the importance of empathy, active listening, and clear communication in fostering a therapeutic environment. Patients often feel vulnerable and anxious when meeting their healthcare provider, making it the provider’s responsibility to remain professional, compassionate, and humane. Such behavior can help address patients’ concerns [2].

Much of what shapes bedside manners today can be traced to the influence of Sir William Osler [3], who emphasized: “Care more for the individual patient than for the special features of the disease. Put yourself in his place… The kindly word, the cheerful greeting, the sympathetic look—these the patient understands…” [3]. This perspective underscores the ethical and emotional aspects of care that extend beyond diagnosis and treatment. Similarly, qualitative perspectives on bedside manners emphasize the experiential, interpersonal, and emotional dimensions of the patient–physical therapist relationship beyond quantifiable outcomes [4].

The significance of emotional connection and compassionate presence in establishing a therapeutic setting is consistently emphasized in qualitative studies, including patient narratives and interviews [5,6]. In a study by Gard [7] it was reported that patients often valued moral considerations more than technical skills and were more engaged in treatment when physical therapists were trustworthy and empathic [7]. Likewise, qualitative interview and focus-group studies indicate that patients are highly aware of their providers’ style of contact, often perceiving hurried or impersonal interactions as barriers to trust and involvement [8,9]. These findings suggest that bedside manners may influence patient satisfaction and clinical outcomes.

In physical therapy, bedside manners assume even greater significance because of the frequency and duration of patient–therapist interactions. Unlike brief clinical encounters, physical therapy often involves ongoing physical contact, trust-building, and responsiveness to individual needs [9]. Studies show that patients value and trust physical therapists who listen attentively, display empathy, provide encouragement, and communicate clearly [8,10,11,12,13]. Evidence further indicates that patients with higher levels of trust in their physical therapist report greater satisfaction, more active participation in treatment, stronger adherence, reduced emotional distress, and better rehabilitation outcomes [8,14,15]. Previous physical therapy literature indicates that clear explanations, opportunities to ask questions, and supportive communication can strengthen patients’ sense of involvement and control during rehabilitation [8,15,16,17]. Conversely, poor or emotionally unsupportive communication may undermine trust and continued engagement with treatment [8,15,16,17]. This further highlights the importance of bedside manners in high-quality clinical care.

Most previous studies examining patient–therapist interactions in physical therapy have relied on qualitative approaches and relatively small samples, limiting the generalizability of their findings to broader populations [7,8,9,15]. Moreover, much of this evidence has been generated outside Saudi Arabia, raising questions about its applicability to the Saudi physical therapy context [5,14]. Given the cultural relevance of communication norms, trust, privacy, and sex-sensitive interactions in Saudi healthcare settings, examining patients’ perceptions of bedside manners may provide context-specific evidence to support patient-centered rehabilitation [18,19]. Although the importance of practitioners’ interpersonal skills is widely recognized, their consistent application may be challenged by factors such as workload and time constraints [20]. Evidence also remains limited regarding how patients themselves perceive these aspects of physical therapy care and their influence on the treatment experience [14,15]. Our previously published companion study examined Saudi physical therapists’ perceptions of bedside manners and demonstrated strong professional support for communication, privacy, reassurance, empathy, and other patient-centered behaviors [21]. However, therapists’ perceptions of appropriate professional behavior may not necessarily reflect patients’ experiences, expectations, or preferences. Therefore, the present study aimed to examine patients’ perceptions of physical therapists’ bedside manners in Saudi Arabia and to explore whether these perceptions varied according to selected patient- and therapist-related characteristics using a mixed-methods approach. Accordingly, the study addressed three research questions: (1) How do patients describe their experiences and preferences regarding physical therapists’ bedside manners? (2) Do selected perceptions and preferences vary according to patient and therapist characteristics, including patient sex, therapist sex, patient–therapist sex match, age, education, and work type? (3) How do patients’ qualitative accounts contextualize the patterns and variability observed in the questionnaire findings, particularly regarding empathy and therapist supervision?

## 2. Materials and Methods

### 2.1. Design

The present patient-focused investigation formed part of the same overarching research program as our previously published study of physical therapists’ perceptions of bedside manners [21], but involved a distinct participant sample and dataset. The study utilized a cross-sectional convergent mixed-methods design in which quantitative and qualitative data were collected during the same overall study period. Neither component was analyzed before completion of data collection, and the qualitative component was not developed in response to preliminary quantitative findings. The quantitative component involved a self-report questionnaire, whereas the qualitative component comprised semi-structured interviews with a self-selected subsample of questionnaire participants. The two components were analyzed separately after data collection and subsequently integrated during interpretation to examine areas of convergence, complementarity, and divergence between questionnaire responses and patients’ qualitative accounts [22].

### 2.2. Participant Recruitment and Sampling

Recruitment took place between June 2024 and November 2024. Participants were recruited using convenience sampling through multiple strategies, including advertisements, word-of-mouth referrals, and collaborations with local and national physical therapy departments and centers. Participants were recruited across all five geographical regions of Saudi Arabia and from different physical therapy settings, including general hospitals, private clinics, and rehabilitation centers; their geographical and facility distributions are reported in Table 1. Recruitment was not proportionate across regions or facility types. Advertisements stated that the study was seeking patient participants. Participants were eligible if they had received physical therapy services within the previous year and had been discharged from treatment during that period. Only patients who had completed their therapy sessions were eligible to participate. Consequently, patients who discontinued physical therapy before completion were not represented, which was recognized as a potential source of selection bias.

A total of 510 participants were recruited. No a priori sample-size calculation was performed; the quantitative sample size was feasibility-based, with recruitment seeking as many eligible participants as practicable during the predefined data-collection period to increase the breadth and precision of the quantitative findings and provide a sufficiently diverse pool for the qualitative component.

From the study sample, 25 participants volunteered to participate in the semi-structured interview phase (Supplementary Table S1). Participants were included based on their willingness to take part in the interviews and their fulfilment of the study eligibility criteria. The qualitative interviews aimed to explore patients’ personal experiences and perceptions of bedside manners in greater depth, while allowing participants to provide detailed reflections on their questionnaire responses. During qualitative data collection, the interview team (S.H.A., M.M.A., R.M.A., J.B.A., F.A.A., W.A.A., R.A., F.M.A.-A., and R.K.A.), under the supervision of S.M.A., met periodically to review the interviews, discuss emerging concepts, and ensure consistency in interview procedures. Recruitment for the qualitative component continued until the team determined that data saturation had been reached, defined as the point at which additional interviews were no longer generating substantively new concepts relevant to the study objectives. This occurred after 25 interviews.

Ethical approval was obtained from the Institutional Review Board (IRB), and the research protocol was reviewed and endorsed by the Research Ethics Committee at Qassim University, Saudi Arabia (IRB 24-91-06; approved 20 May 2024). All adult participants were fully informed of the study’s objectives and procedures and provided written informed consent. For participants younger than 18 years (*n* = 13), written parental or legal-guardian consent was obtained in addition to the participant’s assent before study participation. An online consent form was presented to participants before data collection commenced. Confidentiality was maintained throughout the study through de-identification and the use of unique participant codes.

### 2.3. Data Collection Tools

#### 2.3.1. Quantitative Component

A self-administered questionnaire was developed specifically for this study. It was available in both paper and online formats. The questionnaire contained 25 items: six sociodemographic items (age, patient sex, education, employment status, work type, and therapist sex) and 19 bedside-manner items. The bedside-manner component comprised Section 3.1.1, with nine dichotomous yes/no items, and Section 3.1.2, with ten items rated on a five-point Likert scale. Questionnaire content was developed collaboratively by the research team, drawing on clinical expertise and previous literature concerning bedside manners and patient–therapist interactions [8,15,16,17].

Before data collection, the patient questionnaire underwent a formal relevance assessment [23]. A 23-member relevance panel comprising eight physical therapy academics with more than five years of clinical experience and 15 former recipients of physical therapy independently evaluated each item using a four-point relevance scale ranging from 1 (not relevant) to 4 (highly relevant). Item-level content validity indices (I-CVIs) were calculated across all 23 raters as the proportion assigning scores of 3 or 4, and the scale-level content validity index, calculated using the average method (S-CVI/Ave), was obtained by averaging the item-level indices [23]. I-CVIs ranged from 0.91 to 1.00 and the S-CVI/Ave was 0.93. Although all items exceeded the prespecified 0.78 criterion, feedback resulted in revisions to Section A item 1 and Section B items 3 and 6. Although the relevance-assessment process provided pre-data-collection feedback from both physical therapy academics and former recipients of physical therapy and resulted in revisions to three items, it was not equivalent to a formal pilot study. A separate pilot assessment of questionnaire comprehension, response options, and administration procedures was not conducted. The questionnaire was originally developed and administered in Arabic by a native Arabic-speaking research team. Because Arabic was both the source and administration language, cross-language translation was not required for data collection. English translations of the bedside-manner items are presented in Table 2 and Table 3 for reporting purposes.

The questionnaire addressed various domains such as communication, empathy, professionalism, and responsiveness. The questionnaire was designed as an item-based descriptive instrument rather than as a unidimensional scale intended to generate a total or subscale score. Individual items represented distinct but related components of bedside manners and were analyzed separately; no composite bedside-manners score was calculated. Section A items were analyzed as dichotomous responses, whereas Section B items were reported using their original five-point response categories and collapsed into three directional categories only for the inferential subgroup analyses described in Section 2.4. Because the instrument was not designed as a unidimensional scale, internal consistency was not used to support a total score. Test–retest reliability and construct validity were not evaluated. Example items included: ‘The physical therapist listened to my complaints fully without interrupting me’ and ‘It is necessary for the physical therapist to clarify all aspects of my condition’. Completion of the questionnaire required approximately 5–10 min, and participants were informed of their right to withdraw from the study at any time without consequence. The survey was de-identified at the point of collection: the recruiters did not know the identities of the patients who participated, and participants were not under the care of the recruiters. No direct personal identifiers were recorded.

Among the 13 participants younger than 18 years, nine were aged 15 years or younger and four were aged 16–17 years. For the nine participants aged 15 years or younger, the questionnaire was completed by a caregiver, who was instructed to respond based on direct observations of the interactions between the physical therapist and the child during treatment. The four participants aged 16–17 years completed the questionnaire themselves. The nine caregiver-completed questionnaires were included in the primary quantitative analyses. The age threshold of 15 years for caregiver-completed questionnaires was a pragmatic decision made by the research team rather than a formally validated cutoff.

#### 2.3.2. Qualitative Component

To provide a more comprehensive understanding of patients’ experiences, a self-selected subsample of 25 questionnaire participants took part in semi-structured interviews [24]. Participants could choose to be interviewed in Arabic or English; however, all chose Arabic. Interviews lasted approximately 30–45 min and were conducted by nine trained researchers (S.H.A., M.M.A., R.M.A., J.B.A., F.A.A., W.A.A., R.A., F.M.A.-A., and R.K.A.) under the supervision of S.M.A. Before data collection, all interviewers received training on the study objectives, interview procedures, ethical principles, and the standardized use of the interview guide. Throughout data collection, the qualitative research team held periodic meetings to review interview content, discuss emerging concepts, and maintain consistency in the application of the interview guide. The guide included open-ended questions and probing prompts to encourage participants to describe their experiences freely while allowing interviewers to clarify responses and explore emerging issues in greater depth [25]. The same interview guide and core questions were used across all interviews, with probing questions used as needed to clarify or expand participants’ responses. The original Arabic interview guide and an English translation are provided with the Supplementary Materials. Before each interview, participants were informed about the study purpose, interview procedures, and their rights as research participants. Written informed consent (specific to the semi-structured interview) was obtained before participation, and participants were advised that their involvement was voluntary, that they could withdraw at any time without penalty, and that their responses would be treated confidentially. With participants’ permission, all interviews were audio-recorded to ensure accurate documentation. Interviewees were invited to elaborate on their questionnaire responses and describe their experiences in greater depth. Individual interviews were conducted either face-to-face or remotely via telephone or Zoom, according to participants’ preferences and availability. Although differences in interview mode may have influenced the depth of participants’ responses, the use of a standardized semi-structured interview guide ensured consistency across all interviews.

Following data collection, audio recordings were transcribed verbatim and verified against the original recordings to ensure transcription accuracy. The Arabic transcripts were subsequently translated into English collectively by members of the qualitative research team, all of whom were native Arabic speakers, during their periodic meetings. During these meetings, the team reviewed the translations against the original Arabic transcripts and discussed any ambiguous, context-dependent, or culturally specific Arabic expressions. Where direct translation was unclear, the team discussed the intended meaning and reached consensus on the most contextually appropriate English wording. Participant quotations were lightly edited for grammatical clarity and readability where necessary, without altering their intended meaning. Language-checking software was used only to improve English grammar and readability. After completion of the analysis, all interview recordings and transcripts were stored securely in accordance with the study’s data management procedures.

To protect participant confidentiality, each patient was assigned a unique identification code consisting of a Roman numeral identifying the interviewer, a numerical code representing the physical therapist, and an alphabetical letter identifying the corresponding patient (e.g., x4a). These identifiers were applied consistently throughout transcription, analysis, and reporting, and the same coding system was maintained across both the qualitative and quantitative datasets to facilitate data integration while preserving confidentiality. All members of the qualitative research team, including the supervising senior researcher, were native Arabic-speaking physical therapists with varying levels of clinical and research experience. This shared professional background provided familiarity with the physical therapy context but also had the potential to influence interpretation of participants’ accounts. To minimize role-related and workplace-related influences during recruitment and data collection, none of the qualitative research team members recruited participants from their own workplace, and interviewers had not previously provided clinical care to, supervised, or maintained a personal relationship with their assigned participants. Throughout data collection and analysis, interpretations were discussed collectively during periodic team meetings under the supervision of S.M.A., allowing differing perspectives to be considered and emerging interpretations to be critically reviewed before consensus was reached. During periodic review, the team noted that later interviews were largely confirming previously identified concepts and were not generating substantially new concepts relevant to the study objectives. Based on this assessment, data saturation was considered to have been reached at 25 interviews.

### 2.4. Data Analysis

Quantitative data were analyzed using IBM SPSS Statistics (version 23). Descriptive statistics were used to summarize participants’ characteristics and questionnaire responses. Section A items were dichotomous and showed a pronounced ceiling effect, with affirmative responses ranging from 88.6% to 98.6%; they were therefore reported descriptively only. For inferential analyses, the five-point Likert responses were collapsed into three directional categories—agree (strongly agree/agree), neutral, and disagree (disagree/strongly disagree). This approach combined adjacent response options representing the same directional position while retaining neutral responses as a distinct category, thereby improving interpretability and reducing sparse cells in subgroup contingency tables, consistent with previous physical therapy survey research [26]. Inferential testing was limited to Section B items with sufficient response variability, operationally defined as no single collapsed response category accounting for more than 85% of responses. The 85% threshold was used as a pragmatic analytic screening criterion to avoid multiple subgroup analyses of items with pronounced ceiling effects and was not intended as an established psychometric cutoff. Four items met this criterion (42.4–79% agreement), whereas the remaining items showed ceiling effects (>90% agreement) and were summarized descriptively. Associations between these items and demographic characteristics were examined using Pearson’s chi-square test of independence. Where more than 20% of cells had an expected count below five, Fisher’s exact test with Monte Carlo approximation was used instead. Effect sizes were reported as Cramér’s V. To account for multiple comparisons across the 24 association tests, a Bonferroni-corrected significance threshold was applied (α = 0.05/24 = 0.0021); associations significant at the uncorrected level (*p* < 0.05) but not surviving correction were reported as nominal. A sensitivity analysis excluding the nine caregiver-completed questionnaires was also performed to assess whether proxy responses materially influenced the quantitative findings.

As an additional sensitivity analysis, the four items selected for inferential testing were examined using their original five-point response categories to assess the effect of response categorization on the subgroup analyses.

Patient age, sex, educational level, work type, therapist sex, and patient–therapist sex match were selected as exploratory variables of interest because they could plausibly relate to patients’ expectations and perceptions of clinician communication and interpersonal care. These subgroup analyses were exploratory and unadjusted and were intended to identify bivariate associations rather than estimate the independent effects of individual patient- or therapist-related characteristics. The inferential analyses treated individual patient observations as independent and did not account for potential clustering of patients within therapists or clinical sites.

Qualitative findings from the 25 semi-structured interviews were analyzed using thematic analysis based on Braun and Clarke’s six-phase approach [27]. This involved data familiarization, generation of initial codes, identification of candidate themes, review and refinement of themes, definition and naming of themes, and finally development of the final report. After transcription, the research team repeatedly reviewed the interview material to develop familiarity with participants’ accounts and identify important patterns related to patients’ perceptions. Four researchers (R.M.A., S.H.A., W.A.A., and J.B.A.) independently examined the transcripts and generated initial codes representing relevant concepts within the dataset. The analysis was conducted using the English-translated transcripts, with ongoing reference to the original Arabic versions when needed to ensure that participants’ intended meanings were preserved. The research team subsequently met to compare coding interpretations, discuss areas of similarity and difference, and establish a shared analytical framework. M.M.A. coordinated these discussions and supported the development of the final coding structure, which was further reviewed by the supervising faculty member (S.M.A.) to strengthen the quality and consistency of the analysis. These discussions also provided an opportunity for the researchers to consider how their shared professional backgrounds as physical therapists might influence interpretation of the data. Rather than calculating statistical measures of coding agreement, the researchers used an iterative process of discussion, reflection, and consensus-building to develop the themes. The final themes were refined and defined according to their relevance to the research objectives, resulting in seven overarching themes. Following separate quantitative and qualitative analyses, the two datasets were integrated at the interpretation stage. Integration involved comparing quantitative response patterns with relevant qualitative themes and participant accounts to identify areas of convergence, complementarity, and divergence. For example, qualitative accounts concerning therapist supervision and emotional support were considered alongside the quantitative response patterns for therapist presence and empathy. This integration was used to provide contextual interpretation of the quantitative findings rather than to establish causal explanations.

The trustworthiness of the qualitative analysis was supported through several methodological procedures. Throughout the analysis, the research team held peer discussions with the supervising faculty member (S.M.A.), who provided critical feedback, reviewed emerging interpretations, and encouraged consideration of alternative explanations [28]. An audit trail was maintained to document analytical decisions, changes made during theme development, and the relationship between coded data and final themes [29]. These procedures enhanced transparency and strengthened confidence in the analytical process by demonstrating how the findings were developed from the original interview data.

## 3. Results

A total of 510 participants were included in the study. The sample was predominantly female (60.4%), whereas males comprised 39.6%. Most participants were between 25 and 50 years of age, with those aged 31–40 years representing the largest group (27.6%). Geographically, more than half of the sample was from the Central Region (56.3%), followed by the Eastern (16.7%) and Western (15.9%) regions. Most participants had received treatment in general hospitals (64.1%), with smaller proportions recruited from private clinics (24.7%) and rehabilitation centers (11.2%) (Table 1). Regarding educational background, 63.9% had a university-level education, while 27.8% had completed high school or less. Overall, 63.5% were employed or students. Work categories included other occupations (31.3%), education (16.7%), students (11.6%), and military service (3.9%), while 36.5% reported not working (Table 1).

### 3.1. Quantitative Section

#### 3.1.1. Section A (Yes/No Items)

Across the nine Section A (yes/no) items, affirmative responses ranged from 88.6% to 98.6% (Table 2). The first item assessed familiarity with the term “bedside manners,” whereas the remaining items reflected patients’ reported experiences of therapist behaviors. Among these experience-based items, affirmative responses were highest for the therapist ensuring an appropriate, private environment during the initial assessment (98.6%) and seeking permission before beginning the examination (98.0%), and remained high for listening without interruption (97.6%), reassurance about the condition (97.5%), motivation to continue treatment (96.7%), and therapist self-introduction (94.9%). The least frequently endorsed experience-based item, though still largely positive, concerned involvement in decision-making: 88.6% of patients reported that the therapist sought their opinion about the proposed treatment plan before designing it, while 11.4% did not.

#### 3.1.2. Section B (Likert Scale Items)

Of the ten Section B items, six showed ceiling effects (>90% agreement) and are reported descriptively (Table 3); the remaining four (Q3, Q8, Q9, Q10) had adequate response variability (42.4–79% agreement) and were tested for association with patient sex, therapist sex, patient-therapist sex match, age group, educational level, and work type. Across the 24 tests, effect sizes were uniformly small (Cramér’s V = 0.06–0.17; Supplementary Table S2). After Bonferroni correction (α = 0.05/24 = 0.0021), two associations remained significant, both involving Q8 (preference regarding therapist presence during therapeutic exercise once the patient is proficient). Because Q8 was negatively worded (‘The physical therapist does not need to be present during therapeutic exercises once the patient is proficient’), agreement indicates that therapist presence was considered unnecessary, whereas disagreement indicates a preference for therapist presence. Responses to Q8 differed by the patient-therapist sex match (χ^2^(6) = 29.39, *p* < 0.001, Cramér’s V = 0.17): agreement that the therapist need not be present was highest in male patient/male therapist matches (65/145; 44.8%) and lowest in the two cross-sex matches (11/57 [19.3%] for male therapist/female patient; 8/44 [18.2%] for female therapist/male patient), whereas a preference for the therapist to be present (disagreement) predominated among female patient/female therapist matches (134/264; 50.8%) and both cross-sex matches (approximately 51–53%). Q8 responses also differed by therapist sex (χ^2^(2) = 15.49, *p* < 0.001, Cramér’s V = 0.17), with patients treated by female therapists more often preferring the therapist to be present (164/321; 51.1% disagreed) than those treated by male therapists (63/189; 33.3%) (Supplementary Table S2; Figure 1).

A further set of associations was nominally significant (*p* < 0.05) but did not survive Bonferroni correction. Q8 was associated with patient sex (χ^2^(2) = 12.21, *p* = 0.0022), with female patients more often preferring therapist presence than male patients (156/308 [50.6%] vs. 71/202 [35.1%]). Q8 was also associated with work type (χ^2^(8) = 23.42, *p* = 0.003), with patients working in education showing the highest preference for therapist presence (52/85 [61.2%]). Q9 was associated with work type (χ^2^(8) = 17.87, *p* = 0.022), patient–therapist sex match (χ^2^(6) = 14.43, *p* = 0.025), and patient sex (χ^2^(2) = 6.26, *p* = 0.044, Cramér’s V = 0.11). Acceptance of reduced attention was highest among students (33/59 [55.9%]), whereas patients working in education more frequently rejected reduced attention (38/85 [44.7%]). Acceptance was also highest among male patient/male therapist matches (74/145 [51.0%]), while rejection was highest among female therapist/male patient matches (23/44 [52.3%]). Q3 was associated with patient–therapist sex match (χ^2^(6) = 14.50, *p* = 0.025) and patient sex (χ^2^(2) = 6.77, *p* = 0.034). Agreement was lowest among male patient/male therapist matches (104/145 [71.7%]) and highest among female patient/female therapist matches (217/264 [82.2%]); female patients also showed greater agreement than male patients (253/308 [82.1%] vs. 148/202 [73.3%]). All remaining associations were nonsignificant. Sensitivity analysis excluding the nine caregiver-completed questionnaires produced the same substantive results, including the direction and statistical interpretation of the principal Q8 associations. In an additional sensitivity analysis retaining the original five-point Likert response categories, the two associations that were statistically significant in the primary analysis remained significant: Q8 differed by therapist sex (χ^2^(4) = 25.42, *p* < 0.001) and by patient–therapist sex match (χ^2^(12) = 51.76, *p* < 0.001). These findings indicate that the principal results were robust to the treatment of the Likert responses. The three-category approach, however, was retained as the primary inferential analysis because collapsing adjacent directional response categories reduced sparse cells and provided more stable and interpretable contingency-table comparisons.

### 3.2. Qualitative Section

The thematic analysis of the interview data generated seven themes describing participants’ perceptions and experiences of bedside manners. Each theme is presented below with an interpretive summary supported by representative quotations from participants. Additional participant quotations are presented in Table 4.

#### 3.2.1. Therapeutic Relationship

Participants consistently described bedside manners as central to the therapeutic relationship, associating respect, empathy, active listening, and clear communication with greater comfort, trust, willingness to ask questions, and involvement in care. Poor bedside manners were associated with lower trust and more negative treatment experiences.

vi12a: “*Yes, it is definitely important. If the therapist is respectful, I feel more interested in attending the sessions and more comfortable asking questions about my case. I have had unsuccessful experiences before, and I did not complete treatment because of the therapist*.”

#### 3.2.2. Treatment Adherence

Participants described bedside manner as related to their commitment to treatment. They consistently associated respectful communication, positive attitudes, and supportive interactions with their willingness to attend appointments, continue therapy, and complete their rehabilitation program.

i6a: “*Whether I feel comfortable and respected by the therapist affects whether I complete or discontinue the sessions*.”

Participants perceived treatment adherence as depending not only on the therapist’s clinical competence but also on the quality of interpersonal interactions throughout rehabilitation. Participants associated feeling respected, comfortable, and supported with greater motivation to attend appointments, follow treatment recommendations, and complete their physical therapy sessions. In contrast, participants associated negative attitudes or poor communication with lower motivation, changing therapists, and, in some cases, discontinuing treatment. Some participants perceived continued engagement with therapy as beneficial to their health outcomes.

#### 3.2.3. Nonverbal Communication

Participants consistently highlighted the importance of therapists’ nonverbal communication, describing it as an indicator of the therapist’s interest, professionalism, and commitment to patient care.

xi1b: “*Body language reveals a lot to me. When the therapist behaves exceptionally well, I respond positively; it makes me feel pleased, and I follow the therapist’s instructions correctly. However, if the therapist behaves in the opposite way, I do not respond well, and the treatment plan may not help me*.”

Participants paid close attention to therapists’ body language, including facial expressions, eye contact, smiles, and overall demeanor, using these nonverbal cues to judge whether the therapist was genuinely interested in their care. Participants associated positive nonverbal communication with greater comfort, confidence, cooperation, and engagement. Conversely, negative or indifferent body language was associated with discomfort, lower confidence in the therapist, and decisions about continuing treatment.

#### 3.2.4. Emotional Support

Participants expressed differing views regarding the role of emotional support during physical therapy. While some valued therapists who acknowledged their pain and emotional experiences, others preferred therapists to maintain a professional focus on recovery rather than providing excessive emotional reassurance.

ii1b: “*It is 100% important. When the therapist shows me sympathy, I feel that they understand my suffering, my pain, my helplessness, and my limitations; it gives me a sense of inner comfort*.”

Many participants associated emotional support with comfort, trust, and reassurance throughout rehabilitation. They appreciated therapists who recognized their pain and conveyed understanding of their physical and emotional experiences, describing these interactions as supportive of the therapeutic relationship.

v2b: “*It is very important that the therapist appreciates the patient’s feelings, understands that the patient is in pain, and shows sympathy*.”

One participant distinguished sympathy from understanding, emphasizing the importance of feeling understood rather than pitied.

iv13b: “*I wouldn’t call it sympathy. It is about the therapist making the patient feel understood—showing that they understand what is hurting and recognize the patient’s pain. If the therapist does not convey this feeling, I will have the opposite reaction. If you do not understand my pain, you will not treat me properly. I feel that this is very important: not sympathy, but understanding the patient’s feelings*.”

However, not all participants viewed sympathy as beneficial. Some believed that excessive sympathy could make them feel weak or create unrealistic expectations. These participants preferred therapists who remained encouraging and focused on rehabilitation outcomes while demonstrating understanding without expressing pity. This variation indicates that preferences for emotional support differed among participants.

#### 3.2.5. Therapist Supervision

Many participants valued having the therapist present during physical therapy sessions, particularly during the early stages of rehabilitation when they were still learning how to perform therapeutic exercises correctly.

x15a: “*It is okay for the therapist to leave me alone for a short time during electrotherapy modalities, but the therapist should remain with me while I perform exercises and supervise the treatment sessions*.”

However, some participants believed that the therapist should remain present throughout the entire treatment session, regardless of whether the patient was already familiar with the exercises.

x5b: “*Therapist presence is essential, regardless of whether the patient knows how to perform the exercises, because it motivates the patient*.”

Participants viewed therapist supervision as an important aspect of physical therapy care. They believed that supervision helped ensure exercises were performed correctly, reduced the risk of injury, and increased their confidence during rehabilitation. Many considered close supervision especially important during the initial stages of treatment while learning new exercises. Beyond technical guidance, participants described therapist presence as providing reassurance and motivation, demonstrating interest in the patient’s recovery and enhancing the overall therapeutic experience. While some participants believed supervision could gradually decrease as they became more competent and independent, others preferred continuous therapist presence throughout treatment. Preferences for therapist supervision therefore varied according to perceived competence, confidence, and stage of recovery.

#### 3.2.6. Initial Impression

Participants expressed differing views regarding the importance of the initial impression formed during their first encounter with the physical therapist. While some considered the first interaction essential for establishing comfort and confidence before treatment began, others believed that the therapist’s clinical competence and treatment outcomes were more important than the initial encounter.

ii6a: “*It makes a difference psychologically. A poor first impression creates aversion toward the therapist, and it can be difficult to accept the therapist if their behavior is inappropriate*.”

For many participants, the initial impression established the foundation for the therapeutic relationship. Participants described positive first encounters as supporting psychological comfort, trust, and willingness to engage in treatment, whereas negative first impressions were associated with discomfort and lower confidence in the treatment process. In contrast, other participants placed greater importance on the therapist’s clinical skills and treatment outcomes than on the first interaction. These findings suggest that patients differ in the importance they attach to the initial impression. While some rely on the first encounter to determine whether they feel comfortable beginning treatment, others reserve judgment until they evaluate the therapist’s competence and the effectiveness of the intervention.

x5b: “*I have no concerns about his manner. I came for therapy, and I would not have visited him unless I was confident in his treatment. The ultimate outcome is what matters to me*.”

#### 3.2.7. Professional Consultation

Participants commonly viewed therapists seeking advice or discussing treatment plans with colleagues as a positive aspect of professional practice. They believed that consulting colleagues reflected responsibility, professionalism, and a commitment to providing high-quality patient care.

ii1b: “*It is acceptable to me. It means that the therapist is responsible and professional. Everyone in the workplace has different skills, and asking colleagues for help is professional*.”

Participants generally perceived professional consultation as a strength rather than a sign of inadequate knowledge or competence. They recognized that therapists have different levels of experience and expertise and believed that seeking advice from colleagues demonstrated accountability and dedication to achieving the best possible treatment outcomes. Participants appreciated therapists who were willing to consult others when faced with unfamiliar or complex cases, as this reassured them that clinical decisions were made in their best interests. Participants associated professional consultation with greater confidence in their therapist and with perceptions of professionalism and quality of care.

## 4. Discussion

The main finding of this study is that patients in this sample generally reported positive experiences with their physical therapists’ bedside manners, particularly regarding communication, privacy, consent, reassurance, and professional interpersonal behavior. Although selected perceptions showed differences according to patient- and therapist-related characteristics, only associations involving preferences for therapist presence (Section B, Q8) remained statistically significant after correction for multiple comparisons; the remaining associations were small and are therefore interpreted as exploratory. Overall, the findings suggest that while several core bedside-manner behaviors are consistently valued, preferences regarding some aspects of interpersonal care may vary across patients and according to clinical and sociocultural context. The predominantly positive findings should nevertheless be interpreted in light of the pronounced ceiling effects and the restriction of eligibility to patients who completed treatment, both of which may have favored more positive response patterns. In addition, nine questionnaires (1.8%) were caregiver-completed proxy reports rather than direct patient self-reports; however, sensitivity analysis excluding these responses produced the same substantive quantitative findings.

The quantitative and qualitative findings were complementary rather than directly equivalent. Highly endorsed quantitative behaviors—including communication, privacy, reassurance, and professional conduct—were consistent with qualitative accounts emphasizing respectful communication, nonverbal behavior, emotional support, and the therapeutic relationship. Greater quantitative variability in empathy and therapist presence was similarly reflected in qualitative differences in preferences for emotional support and supervision. These patterns provide contextual understanding of patients’ experiences but do not establish causal relationships with adherence or clinical outcomes [30,31,32,33].

Although patients rated most bedside-manner behaviors very positively, involvement in treatment planning deserves particular attention. This was the least frequently endorsed item in Section A, with 88.6% of participants reporting that their therapist sought their opinion about the proposed treatment program before it was designed, while 11.4% reported that their opinion was not sought (Table 2). This finding is relevant to shared decision-making, which extends beyond providing patients with information or obtaining consent and involves meaningful patient participation in decisions concerning their care. A systematic review of patients’ experiences of shared decision-making in musculoskeletal physical therapy identified trust, effective two-way communication, and opportunities for active patient involvement as important components of the decision-making process [34]. Moreover, observational research in physical therapy has demonstrated that the level of patient involvement achieved during clinical encounters may not always correspond with patients’ preferred level of participation [35]. Therefore, although the overall findings of the present study indicate highly positive perceptions of physical therapists’ bedside manners, the comparatively lower endorsement of involvement in treatment planning suggests that shared decision-making may represent an area in which patient-centered practice could be further strengthened.

Exploratory patterns by patient sex were also observed, although these associations were weak and did not survive correction for multiple comparisons. Female patients showed greater agreement than male patients regarding the importance of empathy (82.1% vs. 73.3%; *p* = 0.034). Although this pattern suggests that perceptions of empathic communication may differ according to patient sex, the small effect size and loss of significance after correction for multiple comparisons warrant cautious interpretation.

Therapist presence was the only survey domain showing demographic associations that remained statistically robust after correction for multiple comparisons. However, both significant associations had small effect sizes (Cramér’s V = 0.17), indicating modest differences in response distributions rather than large or necessarily clinically important differences between groups. Importantly, patients were not uniform in their preferences: 44.5% preferred the therapist to remain present, 31.2% considered therapist presence unnecessary once they were proficient in their exercises, and 24.3% were neutral. The qualitative findings provide further context for this variability, suggesting that supervision serves several functions beyond physical attendance, including ensuring correct exercise performance, reducing perceived risk of injury, providing reassurance, and maintaining motivation. At the same time, some patients considered increasing independence appropriate once exercises had been learned and sufficient confidence had been developed. Therapist presence may therefore be better understood as an individualized and negotiated component of care that varies according to patient competence, confidence, stage of rehabilitation, and personal preference. Because these associations were derived from unadjusted bivariate analyses, they should not be interpreted as independent effects of therapist sex or patient–therapist sex match. Other patient, therapist, treatment, or setting characteristics may have contributed to the observed response patterns.

Although preferences for therapist presence were associated with therapist sex and patient–therapist sex match, the small effect sizes and observational design preclude identifying the mechanism underlying these associations. The qualitative interviews did not identify modesty, sex concordance, or discomfort with cross-sex interactions as explanations. Cultural expectations surrounding privacy and interactions between the sexes may represent one possible interpretation [18,19], but other factors—including therapist behavior, treatment type, diagnosis, clinical setting, patient age, and previous treatment experiences—may also have contributed. Future dyadic research could investigate whether these patterns reflect cultural comfort, perceived therapist behavior, treatment characteristics, or other unmeasured factors.

Responses to Q9 also revealed substantial variation in patients’ expectations regarding attention according to case complexity. While 42.4% of participants considered it acceptable for physical therapists to devote less attention to patients with minor conditions, 37.6% disagreed and 20.0% remained neutral. This relatively even distribution suggests that patients may differ in how they interpret equitable care: some may consider greater attention to more complex cases appropriate, whereas others may expect comparable attention regardless of condition severity. This interpretation should remain cautious, however, because the questionnaire did not distinguish between interpersonal attention and differences in clinical time or resources according to patient need. Exploratory subgroup patterns were also observed, with students more frequently accepting reduced attention and participants working in education more frequently rejecting it; however, these associations did not survive correction for multiple comparisons.

The greater response variability observed for Q3, Q8, Q9, and Q10, together with the diversity of views expressed during interviews, indicates that some aspects of bedside manners may be more dependent on individual preferences and clinical context than consistently endorsed behaviors such as communication, privacy, and consent. The qualitative findings provided additional context by showing how patients related interpersonal and nonverbal behaviors to comfort, engagement, and their overall treatment experience [15,36].

Empathy was strongly endorsed by most participants but produced greater response variability than communication, first impression, or nonverbal behavior. The qualitative findings provide context for this pattern. Patients generally valued being understood and having their pain acknowledged, but some distinguished empathic understanding from excessive sympathy or pity. This distinction suggests that effective empathy in rehabilitation is not simply a matter of displaying more emotion. Rather, patients may value an individualized form of empathy that communicates understanding while preserving autonomy, confidence, and a recovery-oriented focus. Previous rehabilitation research similarly identifies empathy and compassion as important components of therapeutic care and associates perceived empathy with greater patient satisfaction and treatment acceptance [37,38]. The present findings extend this literature by indicating that the preferred expression of empathy may differ among patients and should therefore be responsive rather than formulaic [13].

Professional consultation also emerged as an important aspect of perceived professionalism. In the qualitative interviews, patients generally viewed therapists consulting colleagues about their case as an indication of responsibility and commitment to obtaining appropriate clinical guidance rather than as evidence of incompetence. A related but distinct pattern was observed quantitatively in Q10, where 79.0% of participants agreed that it was acceptable to seek assistance from another physical therapist when they experienced difficulty communicating with their treating therapist, while 12.2% were neutral and 8.8% disagreed. No demographic associations with Q10 were statistically significant. Although these findings involve different circumstances—the qualitative theme concerns therapist-initiated professional consultation, whereas Q10 concerns obtaining assistance from another therapist when the patient experiences a communication difficulty—both suggest that patients are generally receptive to involving additional professional support when needed. These findings should not, however, be interpreted as measuring the same construct.

When considered alongside our previously published study of physical therapists [21], the present findings suggest broadly similar patterns in the importance attributed to communication, privacy, consent, reassurance, and professional interpersonal behavior. Descriptive differences were also apparent in areas such as patient involvement in treatment planning, therapist presence, and attention according to case complexity. However, because the two studies involved independent samples and related but non-identical questionnaire items, these patterns should not be interpreted as evidence of agreement or disagreement between patients and therapists. Rather, they generate hypotheses regarding potential differences between professional and patient perspectives that should be examined using dyadic designs in which both members of the same therapeutic relationship are assessed.

The present findings support the importance of recognizing individual variation in patients’ communication, emotional-support, and supervision preferences. However, the cross-sectional design does not establish that adapting bedside-manner behaviors improves treatment adherence, retention, or clinical outcomes. Similarly, comparisons with our previous therapist study are descriptive because the two studies involved independent samples. Whether greater alignment between patient and therapist preferences improves therapeutic processes or outcomes should be examined prospectively using longitudinal, interventional, and dyadic designs.

## 5. Strengths and Limitations

Major strengths of this study include the relatively large patient sample (*n* = 510) and the integration of quantitative and qualitative methods. Recruitment through multiple physical therapy departments and centers broadened the range of clinical contexts represented, while the semi-structured interviews allowed the survey findings to be interpreted alongside patients’ own accounts of therapeutic interactions. The study also examined therapist sex and patient–therapist sex match, providing additional contextual information on an aspect of physical therapy care that has received comparatively limited attention.

A further strength is the patient-centered focus of the investigation within the Saudi sociocultural context. When considered alongside our previously published companion study of physical therapists [21], the present findings allow for descriptive examination of areas of convergence and divergence between professional and patient perspectives while maintaining the distinction between the two independent samples. The mixed-methods design further strengthens this contribution by showing that highly endorsed behaviors such as communication, privacy, and consent coexist with greater individual variability in preferences regarding empathy, supervision, and attention according to case complexity.

Despite its strengths, the study has several limitations. An important source of potential selection bias is that eligibility was restricted to patients who had completed and been discharged from physical therapy. Patients who discontinued treatment prematurely, including those who may have done so because of dissatisfaction or unfavorable interactions with their therapist, were not represented. Consequently, the predominantly positive responses observed in this study may partly reflect the characteristics of treatment completers and should not be interpreted as representative of all physical therapy patients in Saudi Arabia.

The use of self-reported data introduces potential bias, including inflated positivity, social desirability effects, and subjective interpretations [39]. The high proportion of positive responses across several survey items may have resulted in ceiling effects, with agreement ranging from 88.6% to 98.6%, limiting the ability to detect subtle differences in participants’ perceptions. Although the questionnaire demonstrated strong content validity, this does not preclude ceiling effects or favorable-response bias, and other psychometric properties, including test–retest reliability and construct validity, were not evaluated. Because the instrument was designed for item-level assessment rather than as a unidimensional scale, internal consistency was not used to support a total score; further psychometric evaluation is therefore warranted. The absence of formal pilot testing may also have limited the opportunity to identify difficulties in item comprehension or response interpretation before the main data collection. The cross-sectional design limits causal inference, making it unclear whether the observed associations between demographic factors and perceptions of bedside manners reflect stable patterns or situational variation [39]. In addition, subgroup analyses were based on unadjusted bivariate comparisons, and associations involving therapist sex and patient–therapist sex match may therefore have been influenced by measured or unmeasured confounding factors, including patient characteristics, diagnosis, treatment modality, clinical setting, previous treatment experience, or circumstances associated with therapist assignment. The analyses also did not account for potential clustering of patients within individual therapists or clinical sites, which may have affected the precision of the estimated associations. Accordingly, subgroup findings should be interpreted as exploratory rather than as evidence of independent effects.

The use of convenience sampling may have further introduced selection bias, as individuals with stronger views or greater willingness to discuss their healthcare experiences may have been more likely to participate. Although participants were drawn from all five geographical regions and from several types of physical therapy settings, recruitment was not proportionate across regions or facilities, with the Central Region and general hospitals contributing the largest proportions of the sample. Therefore, the geographic breadth of recruitment should not be interpreted as national representativeness. A small proportion of questionnaires (*n* = 9; 1.8%) were completed by caregivers as proxy respondents, and their perceptions may not fully reflect those of the patients themselves. The decision to use caregiver proxy responses for participants aged 15 years or younger was based on a pragmatic research-team threshold rather than an established validated age cutoff, which may have introduced inconsistency in how younger participants’ perspectives were represented. The qualitative subsample was self-selected, which may have favored participants who were particularly willing to discuss their treatment experiences. The interviews were conducted in Arabic and analyzed using English-translated transcripts, with reference to the original Arabic versions throughout the analytical process. Although translations were collectively reviewed against the original Arabic transcripts and ambiguous or culturally specific expressions were resolved through team discussion and consensus, no formal independent back-translation was performed. Therefore, subtle linguistic or cultural nuances may still have been altered during translation. For the qualitative component, no formal measure of agreement between coders was calculated; to address this limitation, the research team held repeated discussion sessions and reviewed the coding together until consensus was reached on the final themes. The findings should therefore be interpreted with caution and may not be fully generalizable to all physical therapy patients in Saudi Arabia or to other healthcare settings. Despite these limitations, the findings provide valuable insights into patients’ perceptions of physical therapists’ bedside manners and may inform future research and clinical practice.

## 6. Conclusions

In conclusion, this study demonstrated that patients in this sample generally reported highly positive experiences with physical therapists’ bedside manners, particularly regarding communication, attentive listening, consent, privacy, reassurance, and motivation. However, greater variability was observed in perceptions of empathy, therapist supervision, and the allocation of attention according to case complexity, indicating that some aspects of bedside manners may depend more strongly on individual patient preferences and clinical circumstances. After correction for multiple comparisons, the only statistically robust differences concerned preferences for therapist presence according to therapist sex and patient–therapist sex match, although the observed effect sizes were small. The qualitative findings further emphasized the importance of therapeutic relationships, nonverbal communication, emotional support, supervision, and professional consultation in shaping patients’ treatment experiences. These findings suggest that clinicians may benefit from explicitly eliciting individual patient preferences regarding communication, emotional support, and therapist supervision rather than assuming that a single interpersonal approach is appropriate for all patients. However, because this study was cross-sectional and based on self-reported perceptions, it cannot establish that adapting bedside-manner behaviors improves treatment adherence, retention, or clinical outcomes. The effects of individualized communication and supervision strategies should therefore be evaluated prospectively using longitudinal, interventional, and dyadic designs.

## Figures and Tables

**Figure 1:**
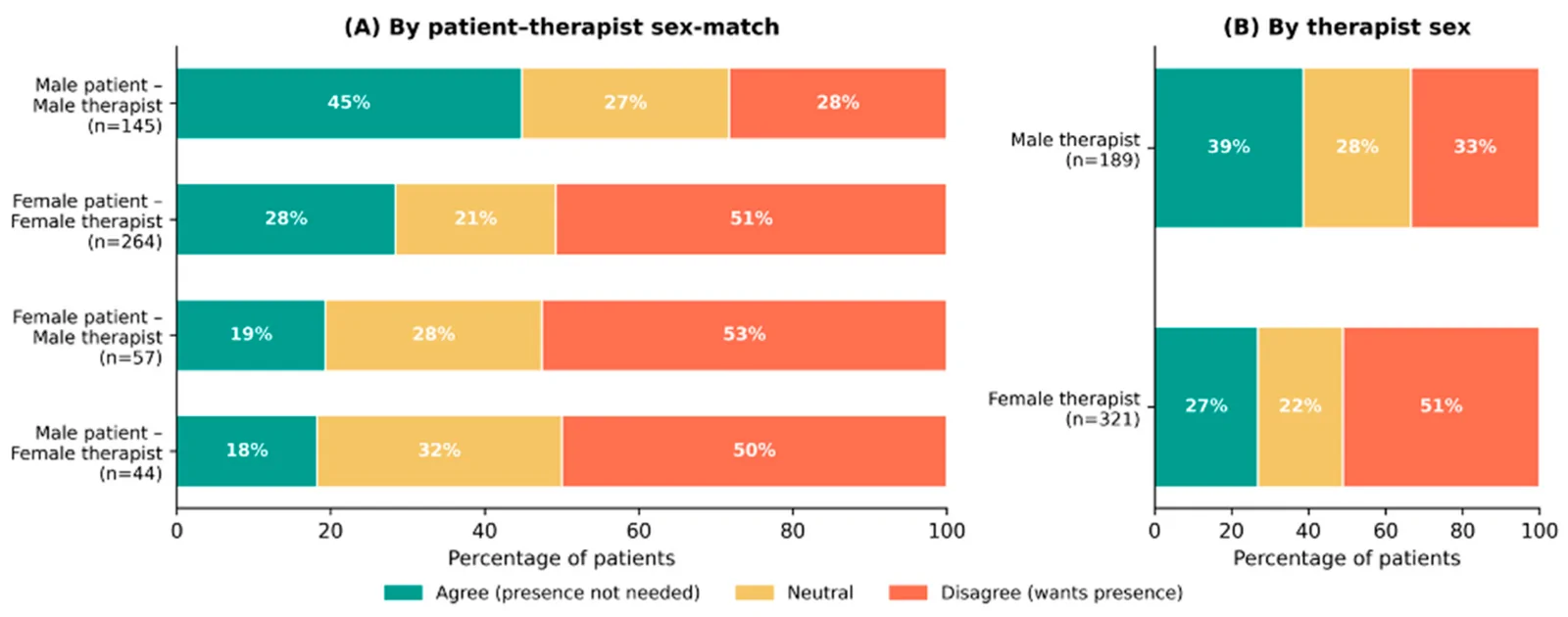
Patient responses to Q8 (preference regarding therapist presence during therapeutic exercise once the patient is proficient), by patient–therapist sex match and therapist sex. Agree = therapist presence not needed; Disagree = therapist presence preferred.

**Table 1:** Patient Demographic Characteristics.

Demographic Characteristics	(*n* = 510)
Age, years	0–17—13 (2.5%)
	18–24—70 (13.7%)
	25–30—108 (21.2%)
	31–40—141 (27.6%)
	41–50—100 (19.6%)
	51–60—59 (11.6%)
	61–80—19 (3.7%)
Geographic Region	Central Region 287 (56.3%)
	Eastern Region 85 (16.7%)
	Western Region 81 (15.9%)
	Northern Region 35 (6.8%)
	Southern Region 22 (4.3%)
Treatment Facility Type	General hospital 327 (64.1%)
	Private clinics 126 (24.7%)
	Rehabilitation center 57 (11.2%)
Patient sex	Female—308 (60.4%)
	Male—202 (39.6%)
Therapist sex	Male—189 (37.1%)
	Female—321 (62.9%)
Patient-therapist sex match	Male patient-male therapist—145 (28.4%)
	Female patient-female therapist—264 (51.8%)
	Female patient-male therapist—57 (11.2%)
	Male patient-female therapist—44 (8.6%)
Education	High school or less—142 (27.8%)
	University level—326 (63.9%)
	Higher education—42 (8.3%)
Employed or student	Yes—324 (63.5%)
	No—186 (36.5%)
Work type	Student—59 (11.6%)
	Education—85 (16.7%)
	Military—20 (3.9%)
	Other—160 (31.3%)
	Not working—186 (36.5%)

**Table 2:** Yes/No Responses to Bedside Manners Questionnaire Section A (Translated).

Items	Yes	No
1-I understand the meaning of the term ‘bedside manners’ or ‘bedside ethics’:	489 (95.9%)	21 (4.1%)
2-During my physical therapy care, the therapist introduced themselves when meeting me for the first time:	484 (94.9%)	26 (5.1%)
3-The physical therapist listened fully to my complaints without interrupting me:	498 (97.6%)	12 (2.4%)
4-During my physical therapy sessions, the therapist consistently sought to reassure me about my condition:	497 (97.5%)	13 (2.5%)
5-During the initial assessment of my health condition, the physical therapist asked for my permission before beginning the physical examination:	500 (98%)	10 (2%)
6-During the initial assessment of my health condition, the physical therapist provided an appropriate environment to ensure privacy during the examination:	503 (98.6%)	7 (1.4%)
7-After completing the examination, the physical therapist fully explained the examination findings and the diagnosis of my condition:	489 (95.9%)	21 (4.1%)
8-Before designing the treatment plan, the physical therapist asked for my opinion about the proposed treatment program for my condition:	452 (88.6%)	58 (11.4%)
9-During the treatment program, the physical therapist consistently motivated me to continue treatment even when there was no noticeable improvement in my condition:	493 (96.7%)	17 (3.3%)

**Table 3:** Patients’ Responses to Bedside Manners Section B Survey (Translated).

Items	Strongly Agree	Agree	Neutral	Disagree	Strongly Disagree
1- The physical therapist should clarify all aspects of my condition, even if it is complex or involves many details:	377 (73.9%)	111 (21.8%)	20 (3.9%)	2 (0.4%)	0 (0.0%)
2- The physical therapist should provide me with a comprehensive medical report about my condition:	307 (60.2%)	167 (32.7%)	29 (5.7%)	7 (1.4%)	0 (0.0%)
3- The physical therapist should show empathy toward my condition, as this is important for the success of the treatment program:	253 (49.6%)	148 (29.0%)	78 (15.3%)	26 (5.1%)	5 (1.0%)
4- The physical therapist should explain all devices and equipment used in my treatment program:	356 (69.8%)	128 (25.1%)	22 (4.3%)	4 (0.8%)	0 (0.0%)
5- A good first impression of the physical therapist is important for the success of the treatment program:	386 (75.7%)	103 (20.2%)	17 (3.3%)	4 (0.8%)	0 (0.0%)
6- The physical therapist’s body language and tone of voice should be considered when interacting with patients:	352 (69.0%)	135 (26.5%)	21 (4.1%)	2 (0.4%)	0 (0.0%)
7- The physical therapist should take into consideration the differences in patients’ age, educational level, and social background:	372 (72.9%)	117 (22.9%)	19 (3.7%)	2 (0.4%)	0 (0.0%)
8- The physical therapist does not need to be present while I perform therapeutic exercises, especially once I am proficient in them:	62 (12.2%)	97 (19.0%)	124 (24.3%)	150 (29.4%)	77 (15.1%)
9- It is acceptable for the physical therapist to give less attention to simple conditions and more attention to difficult or complex conditions:	104 (20.4%)	112 (22.0%)	102 (20.0%)	123 (24.1%)	69 (13.5%)
10- If I experience difficulty communicating with the physical therapist, it is acceptable to seek help from another physical therapist:	169 (33.1%)	234 (45.9%)	62 (12.2%)	32 (6.3%)	13 (2.5%)

**Table 4:** Additional participants’ quotations.

Qualitative Themes	Additional Participant Quotations
Therapeutic Relationship	vi11b: “Honestly, I consider it an essential part of my experience.” v2a: “It is very important, and this is one of the reasons why I may change the therapist or the clinic.” x20g: “It has a major effect. It can influence whether the patient completes treatment, accepts the treatment, and even attends appointments, particularly when the therapist treats the patient well.”
Treatment Adherence	ii1b: “It affects whether I continue attending the sessions and whether I feel satisfied with the progress in my condition.” x2a: “It can lead the patient to change therapists because of the therapist’s attitude. However, when the therapist is respectful, the patient is more likely to continue the intervention and improve their health condition.”
Nonverbal Communication	x2a: “Definitely, I care about the therapist’s smile and cheerfulness, and I consider these characteristics when deciding whether to continue treatment. Even if the therapist is excellent, if they are not cheerful, I may not continue with them.” x26b: “It matters because it gives me an idea of whether the therapist is enthusiastic about the work. Some therapists seem to treat it simply as a job they have to do. This is important because professional ethics go beyond routine work. It gives me a sense of whether the therapist enjoys the profession and is willing to put more effort into it, or whether it is simply a job for which they are paid.”
Emotional Support	vi11b: “It may be important for some people, but for me, the most important thing is that I leave the session feeling better and noticing a difference.” xi2a: “No, sympathy is not necessary in my opinion. The therapist can show some sympathy, but not to the point that it makes me feel weak.”
Therapist Supervision	xi2a: “I think it is necessary for the therapist to remain with the patient throughout the session, even if I know how to perform the exercises. I may perform an exercise incorrectly, and there is a risk of injury.” iv11c: “After some time—around one to two weeks of exercising—it is okay for me to complete the sessions alone.”
Initial Impression	x20g: “If the first interaction was unpleasant, I might excuse it once, but if it happened again, I would think that the therapist’s behavior was inappropriate. Good treatment might compensate for that to some extent. I came for therapy, not to socialize, but if my condition is not improving as expected, I think it is reasonable to change therapists.” iv11c: “For me, the first impression has a strong influence on the treatment experience.”
Professional Consultation	x20g: “I have no problem with it. It means that the therapist is seeking accurate information and the best approach for the patient to avoid taking an inappropriate step. I may want to know why the therapist is asking another specialist for help, but if the therapist is unsure about something, seeking advice shows that they are looking for the best treatment for the patient.” x26b: “It is okay; I have no problem with it. To me, it shows that the therapist cares and is trying to provide the best service. I do not think it means that the therapist is unqualified; rather, it shows that they are trying to do their best.”

## Data Availability

The data presented in this study are available on request from the corresponding author due to privacy concerns, as the dataset contains potentially identifying participant information.

## Supplementary Materials

The following supporting information can be downloaded at: https://www.mdpi.com/article/10.3390/healthcare14193231/s1, Table S1: Characteristics of the interviewed patients (*n* = 25); Table S2: Associations between Section B items with adequate variability (Q3, Q8, Q9, Q10) and demographic characteristics; Table S3: Cross-tabulation of Q8 (need for therapist presence during the session) by demographic characteristics, *n* (%); Table S4: Joint display integrating key quantitative findings with qualitative themes; Supplementary File S1: Original Arabic semi-structured interview guide; Supplementary File S2: English translation of the semi-structured interview guide.
