# Supplementary material for: Patients’ Perspectives on Bedside Manners in Physical Therapy in Saudi Arabia: A Mixed-Methods Study
Source: Healthcare (Basel). 2026 Oct 1;14(19):3231. doi: 10.3390/healthcare14193231 (PMC13664845; doi:10.3390/healthcare14193231)
Supplement: Supplementary file 1 [file healthcare-14-03231-s001.zip › healthcare-4555008-supplementary.pdf]

Supplementary Table S1. Characteristics of the interviewed patients (n = 25).

ID	Age	Patient sex	Education	Employed/Student	Work type	Therapist sex
i6a	37	F	University	No	Not Working	F
i7a	49	F	Secondary School or Less	No	Not Working	F
ii1b	36	F	University	Yes	Education	F
ii6a	34	F	University	Yes	Student	F
iv11c	32	F	University	Yes	Other	F
iv13b	30	M	University	Yes	Other	F
v2a	42	M	Secondary School or Less	Yes	Military	F
v2b	40	F	University	No	Not Working	F
vi11b	42	M	Higher Education	Yes	Other	F
vi12a	23	F	University	Yes	Student	M
viii17a	21	M	University	Yes	Student	M
viii18a	32	F	Secondary School or Less	No	Not Working	F
ix1a	44	F	University	No	Not Working	M
ix1b	38	F	Higher Education	No	Not Working	M
x2a	24	M	Secondary School or Less	Yes	Military	M
x5b	22	M	University	Yes	Other	M
x7f	30	M	University	Yes	Other	M
x13a	44	F	University	Yes	Other	F
x14e	44	F	University	No	Not Working	F
x15a	27	F	University	Yes	Other	F
x16c	28	F	University	No	Not Working	F

x20g	20	M	University	Yes	Student	M
x26b	42	F	University	Yes	Other	M
xi1b	34	M	Higher Education	Yes	Other	F
xi2a	47	F	University	No	Not Working	F

ID: consists of a Roman numeral identifying the interviewer, a numerical code representing the physical therapist, and an alphabetical letter identifying the corresponding patient; F = female, M = male. Therapist sex denotes the sex of the physical therapist who treated each interviewed patient.

Supplementary Table S2. Associations between Section B items with adequate variability (Q3, Q8, Q9, Q10) and demographic characteristics.

Item	Demographic	χ^2	df	p	Cramér's V	Test
Q3	Patient sex	6.77	2	0.034	0.12	χ^2
Q3	Therapist sex	4.53	2	0.104	0.09	χ^2
Q3	Patient–therapist sex-match	14.50	6	0.025	0.12	χ^2
Q3	Work type	8.56	8	0.381	0.09	χ^2
Q3	Education	7.80	4	0.099	0.09	χ^2
Q3	Age group	15.85	12	0.198	0.12	Fisher/MC
Q8	Patient sex	12.21	2	0.0022	0.15	χ^2
Q8	Therapist sex	15.49	2	<.001	0.17	χ^2
Q8	Patient–therapist sex-match	29.39	6	<.001	0.17	χ^2
Q8	Work type	23.42	8	0.003	0.15	χ^2
Q8	Education	5.49	4	0.240	0.07	χ^2
Q8	Age group	20.06	12	0.066	0.14	χ^2
Q9	Patient sex	6.26	2	0.044	0.11	χ^2
Q9	Therapist sex	1.69	2	0.430	0.06	χ^2
Q9	Patient–therapist sex-match	14.43	6	0.025	0.12	χ^2

Q9	Work type	17.87	8	0.022	0.13	χ^2
Q9	Education	4.21	4	0.378	0.06	χ^2
Q9	Age group	18.67	12	0.097	0.14	χ^2
Q10	Patient sex	2.23	2	0.329	0.07	χ^2
Q10	Therapist sex	4.85	2	0.088	0.10	χ^2
Q10	Patient–therapist sex-match	9.72	6	0.137	0.10	χ^2
Q10	Work type	10.10	8	0.258	0.10	χ^2
Q10	Education	3.31	4	0.507	0.06	χ^2
Q10	Age group	11.10	12	0.520	0.10	χ^2

Bonferroni-corrected threshold α = .05/24 = .0021. Fisher/MC = Fisher's exact test with Monte Carlo approximation, used where >20% of cells had expected counts <5.

Supplementary Table S3. Cross-tabulation of Q8 (need for therapist presence during the session) by demographic characteristics, n (%).

Characteristic	n	Agree	Neutral	Disagree
**Patient sex**				
male	202	76 (37.6%)	55 (27.2%)	71 (35.1%)
female	308	83 (26.9%)	69 (22.4%)	156 (50.6%)
**Therapist sex**				
Male	189	73 (38.6%)	53 (28.0%)	63 (33.3%)
Female	321	86 (26.8%)	71 (22.1%)	164 (51.1%)
**Patient–therapist sex-match**				
Both males	145	65 (44.8%)	39 (26.9%)	41 (28.3%)
Both females	264	75 (28.4%)	55 (20.8%)	134 (50.8%)
Female patient–male therapist	57	11 (19.3%)	16 (28.1%)	30 (52.6%)
Male patient–female therapist	44	8 (18.2%)	14 (31.8%)	22 (50.0%)

Work type				
Student	59	20 (33.9%)	18 (30.5%)	21 (35.6%)
Education	85	18 (21.2%)	15 (17.6%)	52 (61.2%)
Military	20	8 (40.0%)	5 (25.0%)	7 (35.0%)
Other	160	53 (33.1%)	50 (31.3%)	57 (35.6%)
Not working	186	60 (32.3%)	33 (17.7%)	93 (50.0%)

Agree = therapist presence not needed; Disagree = therapist presence preferred.

Supplementary Table S4. Joint display integrating key quantitative findings with qualitative themes.

Quantitative finding	Related qualitative theme	Integrated interpretation
Core communication/privacy/consent items were highly endorsed	Therapeutic Relationship; Nonverbal Communication	Both components emphasized respectful communication and interpersonal behavior as central to patients' experiences
Q3 empathy showed greater response variability	Emotional Support	Interviews indicated that patients valued understanding but differed regarding sympathy or emotional reassurance
Q8 therapist presence showed substantial variability	Therapist Supervision	Interviews suggested differing preferences for continuous supervision versus increasing independence
Q9 responses were divided regarding attention according to case complexity	Therapeutic Relationship / Professional expectations	Patients differed in expectations regarding how attention should be distributed according to clinical complexity
Q10 indicated broad acceptance of assistance from another therapist	Professional Consultation	Both findings reflected openness to additional professional input, although the quantitative and qualitative components addressed distinct clinical situations

Supplementary File S1: Original Arabic Semi-Structured Interview Guide

دليل المقابلة (مقابلة شبه منظمة)

سؤال البحث:

كيف يدرك المرضى ويصفون أسلوب تعامل أخصائيين العلاج الطبيعي معهم — بما في ذلك التواصل، والتعاطف، والاهتمام، والسلوك الشخصي — ودور ذلك في تجربة العلاج الطبيعي؟

قبل البدء بالمقابلة:

- التعريف بالنفس، وتقديم ملخص موجز، وتوضيح هدف الدراسة.
- شكر المشارك على موافقته على المشاركة في الدراسة.
- إتاحة الوقت لطرح المزيد من الأسئلة والإجابة عن أي استفسارات تتعلق بالبحث قبل المضي قدماً.
- التأكد من قراءة وتوقيع المشارك على ورقة معلومات المشارك ونموذج الموافقة.
- التأكيد مجدداً بأن جلسة المقابلة سيتم تسجيلها صوتياً، وطمأنة المشارك بشأن السرية.

الأسئلة المحورية للمقابلة:

- **ما هو مفهومك لأخلاقيات التعامل السريري وتعامل الاخصائي مع المريض اثناء الجلسة العلاجية؟**
  - ماذا تعني لك أخلاقيات التعامل السريري
- **هل تعتقد أن اخلاقيات التعامل مع المريض التي يظهرها الاخصائي لك اثنا الجلسة العلاجية مهمه؟**
  - كيف تصف أهميتها؟
- **هل ترى أن اخلاقيات الاخصائي قد توثر على نتائج العلاج أو تحسن الحالة؟**
  - كيف يمكن أن تؤثر على تجربتك العلاجية؟
- **هل برأيك ان من الطبيعي الاخصائي يعطيك اهتمام اعلى او اقل بحسب صعوبة او سهولة حالتك؟**
  - كيف ترى مستوى الاهتمام الذي ينبغي أن يقدمه الأخصائي للمرضى؟
- **كيف ممكن أن تؤثر طريقة تواصل الأخصائي معك اثناء الجلسة العلاجية على تجربتك؟**
  - أسلوب حديثه، ووضوح شرحه، واستماعه لك، وإعطائك فرصة للتعبير عن رأيك، ولغة جسده ونبرة صوته؟
- **هل برأيك اظهار تعاطف الاخصائي مع المريض اثناء الجلسة العلاجية مهم؟**
  - كيف ترى دورها أثناء الجلسة العلاجية؟

أسئلة المتابعة:

يمكن للباحث استخدام أسئلة المتابعة التالية مع أي من الأسئلة الرئيسية، حسب طبيعة إجابة المشارك، دون الحاجة إلى طرح جميع الأسئلة:

- هل يمكنك أن توضح أكثر؟
- هل يمكنك إعطائي مثالًا؟
- كيف جعلك ذلك تشعر؟
- كيف أثر ذلك على تجربتك العلاجية؟
- كيف أثر ذلك على علاقتك بالأخصائي؟
- هل هناك جانب آخر مهم يتعلق بهذا الموضوع تود إضافته؟

إنهاء المقابلة

اسأل المشارك عما إذا كان يرغب في إضافة أي شيء للمقابلة، واشكره مجدداً على وقته.

Supplementary File S2: English Translation of the Semi-Structured Interview Guide

* The following is an English translation of the original Arabic semi-structured interview guide used in this study. The English version is provided for reporting and methodological transparency and was not used for data collection.

Interview Guide (Semi-Structured)

Research Question

How do patients perceive and describe physical therapists' bedside manners, including communication, empathy, attention, and interpersonal behavior, and their role in the physical therapy treatment experience?"

Before Beginning the Interview

- Introduce yourself, provide a brief overview, and explain the purpose of the study.
- Thank the participant for agreeing to participate in the study.
- Allow the participant sufficient time to ask additional questions and address any concerns related to the research before proceeding.
- Confirm that the participant has read and signed the participant information sheet and consent form.
- Reconfirm that the interview session will be audio-recorded and reassure the participant regarding confidentiality.

Core Interview Questions

- **What is your understanding of bedside manners and the way the physical therapist interacts with the patient during the treatment session?**

  - What do bedside manners mean to you?

2. Do you think that the bedside manners demonstrated by the physical therapist during the treatment session are important?

- How would you describe their importance?

3. Do you think that the physical therapist's bedside manners can affect treatment outcomes or improvement in the patient's condition?

- How might they affect your treatment experience?

4. Do you think it is appropriate for a physical therapist to give you more or less attention depending on the severity or complexity of your condition?

- How do you view the level of attention that a physical therapist should provide to patients?

5. How might the way the physical therapist communicates with you during the treatment session affect your experience?

- Consider the therapist's manner of speaking, clarity of explanation, listening to you, giving you an opportunity to express your opinion, body language, and tone of voice.

6. Do you think it is important for the physical therapist to show empathy toward the patient during the treatment session?

- How do you view the role of empathy during the treatment session?

Follow-Up Questions

The researcher may use the following follow-up questions with any of the main questions, depending on the participant's response. It is not necessary to ask all of them:

- Could you explain further?
- Could you give me an example?
- How did that make you feel?
- How did that affect your treatment experience?
- How did that affect your relationship with the physical therapist?
- Is there any other important aspect related to this topic that you would like to add?

Ending the Interview

Ask the participant whether they would like to add anything else to the interview, and thank them again for their time.
